# Cannabinoid and Terpenoid Doses are Associated with Adult ADHD Status of Medical Cannabis Patients

**DOI:** 10.5041/RMMJ.10384

**Published:** 2020-01-30

**Authors:** Jeffrey Y. Hergenrather, Joshua Aviram, Yelena Vysotski, Salvatore Campisi-Pinto, Gil M. Lewitus, David Meiri

**Affiliations:** Faculty of Biology, Technion–Israel Institute of Technology, Haifa, Israel

**Keywords:** ADHD, cannabis, cannabinoids, terpenes

## Abstract

**Objective:**

The aim of this cross-sectional questionnaire-based study was to identify associations between the doses of cannabinoids and terpenes administered, and symptoms of attention deficit hyperactivity disorder (ADHD).

**Methods:**

Participants were adult patients licensed for medical cannabis (MC) treatment who also reported a diagnosis of ADHD by a physician. Data on demographics, ADHD, sleep, and anxiety were collected using self-report questionnaires. Data collected on MC treatment included administration route, cultivator, cultivar name, and monthly dose. Comparison statistics were used to evaluate differences in reported parameters between low (20–30 g, *n*=18) and high (40–70 g, *n*=35) MC monthly dose and low adult ADHD self-report scale (ASRS, 0–5) score (i.e. ≤3.17 score, *n*=30) or high ASRS score (i.e. ≥3.18 score, *n*=29) subgroups.

**Results:**

From the 59 patients that answered the questionnaire, MC chemovar could be calculated for 27 (45%) of them. The high MC monthly dose group consumed higher levels of most phyto-cannabinoids and terpenes, but that was not the case for all of the cannabis components. The high dose consumers and the ones with lower ASRS score reported a higher occurrence of stopping all ADHD medications. Moreover, there was an association between lower ASRS score subgroup and lower anxiety scores. In addition, we found an association between lower ASRS score and consumption of high doses of cannabinol (CBN), but not with Δ-9-tetrahydrocannabinol (THC).

**Conclusion:**

These findings reveal that the higher-dose consumption of MC components (phyto-cannabinoids and terpenes) is associated with ADHD medication reduction. In addition, high dosage of CBN was associated with a lower ASRS score. However, more studies are needed in order to fully understand if cannabis and its constituents can be used for management of ADHD.

## INTRODUCTION

Attention deficit hyperactivity disorder (ADHD) is a common, heritable, neuropsychiatric disorder affecting 2.5%–5% of adults.^1^,^2^ It is described as a neurodevelopmental syndrome that emerges in childhood or early adolescence; in 60%–70% of cases it persists into adulthood.^3^–^5^ It is characterized by symptoms of inattention or hyperactivity, and impulsivity, or both.^6^ These core symptoms typically manifest as restlessness, mind-wandering, emotional instability, and an inability to relax or concentrate.^7^ Lower educational attainment and lower levels of employment are also reported in patients with adult ADHD.^8^ Psychiatric conditions such as depression, anxiety, substance abuse disorder, and antisocial disorders are common psychiatric comorbidities in ADHD.^9^,^10^ The neurobiology and brain circuitry of both ADHD and other comorbid psychiatric disorders are reported as being similar.^11^ A large body of evidence reveals that untreated adult ADHD leads to various negative psychosocial consequences.^6^ Effective treatment can help prevent these negative outcomes.^12^

The management of ADHD typically includes psychostimulant medications (methylphenidate and amphetamine derivatives),^13^ non-stimulant medications (e.g. atomoxetine),^14^ and extended-release clonidine and guanfacine.^15^ Multiple other medications are used “off-label,” with less efficacy and tolerability.^15^ Nonetheless, methylphenidate remains the most prescribed, efficacious, and tolerated medication for ADHD.^13^,^15^ The non-serious adverse effects (AEs) of these medications include insomnia, decreased appetite, anxiety, increased systolic and diastolic blood pressure,^16^,^17^ nausea, dry mouth, fatigue, headache, urinary hesitation, erectile dysfunction,^18^ infection, and nervousness.^19^ A thorough review of the safety of approved ADHD medication has been conducted elsewhere.^20^

Increasingly, there is recognition that medical cannabis (MC) may offer an alternative treatment option for adult ADHD.^21^ In one case report, treatment with MC revealed marked improvement of ADHD symptoms.^22^ In addition, an uncontrolled collection of clinical case reports from 30 treatment-resistant ADHD patients reported MC to be an effective and well tolerated treatment.^23^ In contrast, the first and only randomized controlled trial of 30 participants using nabiximols, a balanced extract of Δ-9-tetrahydrocannabinol (THC) and cannabidiol (CBD), showed no statistically significant reduction of ADHD symptoms. Notably, no ADHD symptoms worsened.^7^ It is yet to be elucidated, in a controlled manner, whether other combinations of cannabinoids (and terpenoids) are capable of reducing symptoms in ADHD.

Currently there is a gap in the literature concerning the clinical effects of the specific cannabis plant cannabinoid and terpenoid components, best termed “chemovars” rather than utilizing “strain names,” otherwise termed “cultivars.”^24^ Thus far, many specific phyto-cannabinoids^25^ and many terpenoids^26^ have been identified and quantified, making it possible to use this information in clinical trials. However, current studies on ADHD and MC disregarded MC treatment complexity, and evaluated it as if it was a single compound.^21^ In reality, patients consume combinations of cannabis cultivars, tailoring their own specific treatment by trial and error, making dosing of cannabinoid and terpenoid constituents different for each patient. In Israel, ADHD is not a qualifying condition for MC treatment.^27^ However, of the 51,000 patients in Israel currently approved for MC treatment, a significant cohort report a comorbidity of ADHD.

Medical cannabis in Israel is governed by the Israeli Ministry of Health (IMOH) under regulations of cannabis use for medical purposes. There are specific indications for which a physician can request a license for a patient; ADHD is not a qualifying condition for a MC license. However, it is a comorbidity of some patients with an approved indication (e.g. chronic pain, gastrointestinal disease, etc.). Generally, a MC application is received by one of the board members of the Medical Cannabis Unit (MCU) that would reply to the physician if the request is approved or refused, and the reason for the refusal.

Physicians in Israel decide in collaboration with the patient on the route of administration that is approved by the MCU, either inflorescence for smoke and vaporization, and/or oil extracts for sublingual use. The monthly dose of MC is decided by the physician (starting monthly dose is generally indicated as 20 g by the MCU; any increase is also subject to MCU approval). Physicians provide consultation for the selection of specific MC cultivar or combination of cultivars. However, the final decision on the selection of MC cultivar(s) is in the hands of the patient. Hence, the consumption of cannabinoid or terpenoid doses is not controlled. Every patient goes through a personal trial-and-error process to find the cultivar or the combination of cultivars that best meets his/her therapeutic needs. Moreover, instructions for titration (starting dose, doses per day, guidelines for increasing/decreasing of the dose, or maximum dose allowed) of MC treatment are made either by a nurse in some centers, but mostly by instructions provided by one of the nine licensed suppliers, eight of which are cultivators. Importantly, these guidelines are only recommendations and are not enforced.

The purpose of this cross-sectional study was to examine the differences between MC monthly dose and ADHD symptoms frequency scores subgroups of ADHD patients, their specific chemovar consumption, and ADHD medication use. Additionally, sleep and anxiety symptoms were evaluated as well as MC treatment AEs.

## MATERIAL AND METHODS

### Subjects

Participants were eligible to participate if they were Hebrew-speaking, aged ≥18 years, reported a diagnosis of ADHD by a physician, and had a standing MC license for the treatment of any approved condition.

All participants received written explanation of the study via email prior to their enrollment. They were asked to sign an electronic informed consent form before the start of data collection. Only patients that signed the informed consent form were allowed to participate in the survey. The study was approved prior to data collection by the institutional ethics committee of the Technion (# 011-2016).

### Study Procedure

The data for this cross-sectional study were collected from an existing database of Israeli patients with a pre-existing MC license for various indications (*n*=3,218). Patients that reported having a diagnosis of ADHD (*n*=367, 11%), and who had previously agreed electronically to disclose their email address for future studies, received an email with an explanation of the study and a link to the online questionnaires. Participants who signed the electronic consent form, and confirmed their ADHD was diagnosed by a physician, were invited to complete the questionnaires. Data were collected in October 2019 to January 2020. No financial compensation was offered to participating patients. Additionally, most clinically administered cultivars of all eight approved cultivators in Israel were evaluated in the laboratory for their cannabinoid (by liquid chromatography–mass spectrometry [LC-MS]) and terpenoid (by gas chromatography–mass spectrometry [GC-MS]) presence and quantity.

### Online Survey

Data collection was carried out online by secure survey technology Qualtrics® (version 12018; Provo, UT, USA).^28^

### Study Questionnaires

Questionnaires collected demographic information that included age, gender, education, body mass index (BMI), and approved MC treatment duration (years). Data on ADHD comorbidities included the past or present diagnosis of: clinical depression, anxiety disorders, antisocial disorders, bipolar disorder, dyslexia, substance abuse, alcohol abuse, and the past or present pharmaceutical treatment of at least one of these comorbidities. Validated questionnaires included the adult ADHD self-report scale (ASRS-v1.1),^29^ the ADHD rating scale,^30^ the Pittsburgh Sleep Quality Index (PSQI),^31^ and the General Anxiety Disorder (GAD-7) scale.^32^ Participants also reported on their MC treatment characteristics and related adverse effects, including administration route, cultivator brand, cultivar name, total monthly dose (g), and monthly dose of each specific cultivar named (g).

### Phyto-cannabinoid Identification and Quantification by LC-MS

Phyto-cannabinoid analyses were performed using a Thermo Scientific ultra-high-performance liquid chromatography (UHPLC) system coupled with a Q Exactive™ Focus Hybrid QuadrupoleOrbitrap mass spectrometer (MS) (Thermo Scientific, Bremen, Germany).^33^ Identification and absolute quantification of phyto-cannabinoids were performed by external calibrations.^34^

### Terpenoid Identification and Quantification by GC-MS/MS

Terpenoid analyses were performed on a Trace 1310 gas chromatography (GC) system (Thermo Scientific) coupled to a TSQ 8000 Evo triple-quadrupole MS (Thermo Scientific) equipped with a DB-35MS UI capillary column (30 m × 0.25 mm × 0.25 μm, Agilent, Santa Clara, CA, USA) as described elsewhere.^35^

### Statistical Analysis

R software (V.1.1.463) with tidyverse^36^ and atable^37^ packages were used to analyze differences in outcome measures by Pearson chi-square for categorical measures and Kruskal–Wallis rank sum test for numeric measures. For effect size (i.e. odds ratio [OR]) and CI we utilized Cohen’s *d* test.^37^ In an attempt to diminish the variability between the cultivars that were analyzed in our lab and those utilized by the patients, only phyto-cannabinoids that were consumed at above a median of 0.1 g per month and terpenoids above a median of 400 parts per million were analyzed. The specific cannabinoid and terpenoid monthly doses were calculated for each patient. Only calculations of the decarboxylated cannabinoids of inflorescence (without oil extracts) were utilized. The MC monthly dose was grouped into low (i.e. 20–30 g) and high dose (i.e. 40–70 g) subgroups. The frequency of ADHD symptoms, reported as a total score (1–5 score range) on the ASRS questionnaire, was also grouped into low ASRS score (i.e. better; ≤3.17 score with *n*=30) or high ASRS score (i.e. worse; ≥3.18 score with *n*=29) subgroups. We analyzed the differences of all measures between MC monthly dose subgroups and ASRS subgroups. Notably, due to missing data (*n*=6) on MC monthly dose, demographic data are presented only for patients with that information. The Shapiro–Wilk test of normality demonstrated non-normal distribution for all measures, thus, data are presented as median and quartiles 25 and 75 (Q1–Q3, IQR). Differences were considered significant at the *P*<0.05 level, after Bonferroni corrections. Incidences are presented as number and percentage of patients.

## RESULTS

### Subjects

**Figure 1:**
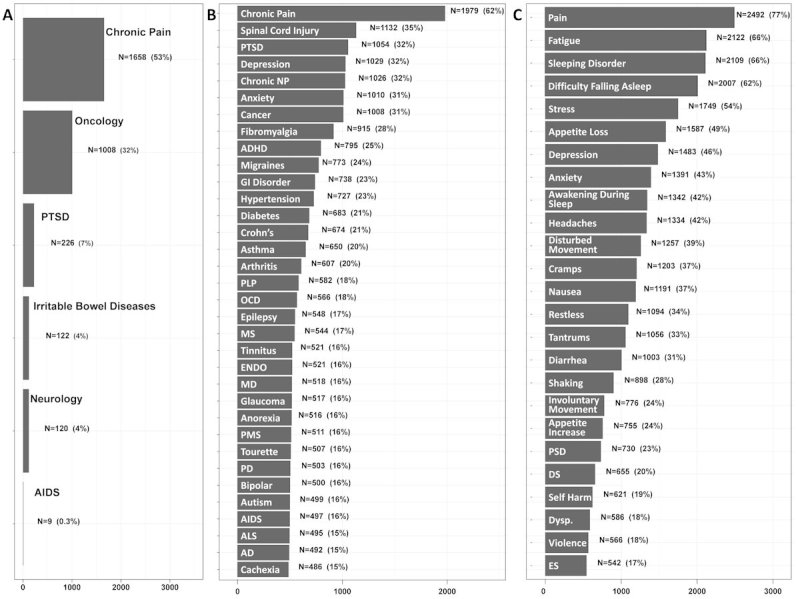
Database Population Clinical Characteristics with the Number of Patients and Percentage Displayed. A: Approved indications for medical cannabis (MC) treatment (total *n*=3,143); B: Comorbidities (total *n*=3,218); and C: Comorbidities symptoms (total *n*=3,218). AD, Alzheimer’s disease; ADHD, attention deficit hyperactivity disorder; AIDS, acquired immune deficiency syndrome; ALS, amyotrophic lateral sclerosis; DS, disturbed speech; Dysp., dysphagia; ENDO, endometriosis; ES, epileptic seizures; GI, gastrointestinal; MD, muscular dystrophy; MS, multiple sclerosis; NP, neuropathic pain; OCD, obsessive compulsive disorder; PD, Parkinson’s disease; PLP, phantom limb pain; PMS, post-menstrual syndrome; PSD, preserved sleep duration; PTSD, post-traumatic stress disorder.

We established a patient-reported outcomes database of Israeli patients with a pre-existing MC license for various MCU-approved indications (*n*=3,218), including chronic pain (*n*=1,658, 53%), oncology (*n*=1,008, 32%), post-traumatic stress disorder (*n*=226, 7%), irritable bowel diseases (*n*=122, 4%), neurological disorders (*n*=120, 4%), and acquired immune deficiency syndrome complications care (*n*=9, 0.3%). Data were missing for 75 patients. This population reported on a variety of comorbidities and associated symptoms (Figure 1). The database population consisted of mostly males (*n*=1,850, 57%), aged 43 (33–56) years.

In this database population, 367 patients (11%) reported having a diagnosis of ADHD. Of them, 110 patients responded to our invitation to participate in the study (30% response rate). Upon confirmation that a physician diagnosed them with ADHD (*n*=80), they were directed to complete the questionnaires. A total of 59 patients answered the study questionnaires, from which, 53 patients reported their MC monthly doses. Notably, most (*n*=47, 89%) patients consumed inflorescences, either by smoking, vaporizing, or both. Five (9%) patients combined oil extracts and inflorescences, and one (2%) patient consumed only oil extracts. Medical cannabis treatment duration of our sample ranged from 1 to 16 years. The MC chemovar constituents dose consumption (i.e. cannabinoid and terpenoid amounts) could be calculated for 27 (50%) of the patients that consumed only MC inflorescence and reported fully on their MC treatment regimen.

### Medical Cannabis Treatment Characteristics

The high MC dose (g) subgroup (i.e. 40–70 g, *n*=35) consumed MC more frequently, with a median of 6 (4–12) times per day, while the low MC dose subgroup (i.e. 20–30 g, *n*=18) consumed MC 3 (2.2–5.8) times per day (OR 0.85; 95% CI −1.5 to −0.23; *P*<0.05). Sample demographics were similar for both MC dose subgroups, the subgroups consisting of a majority of females (*n*=31, 58%), with median age of 38 years (31–46) (Table 1). Notably, anxiety scores, sleep quality, sleep latency, and sleep duration did not vary significantly between the MC dose subgroups.

**Table 1:** Demographic Characteristics.

Measure	Low MC Dose (20–30 g) *n*=18	High MC Dose (40–70 g) *n*=35	Statistic (*P*)	OR (95% CI)
Gender, *n* (%)				
Male	6 (33)	16 (46)	0.16* (0.57)	1.7 (0.45, 6.7)
Female	12 (67)	19 (54)		

Age (years), median (IQR)	38 (32–45)	38 (30–47)	0.16† (0.97)	0.2 (−0.45, 1.86)

BMI, median (IQR)	25 (22–27)	23 (20–27)	0.26† (0.40)	0.1 (−0.48, 0.69)

Education, *n* (%)				
High school	7 (39)	9 (26)	0.45* (0.50)	0.55 (0.14, 2.2)
Higher education	11 (61)	26 (74)		

Employment status, *n* (%)‡				
Full time	7 (39)	16 (46)	0.03* (0.86)	0.76 (0.2, 2.8)
Part time	6 (33)	11 (31)	0* (1)	1.1 (0.26, 4.2)
Unemployed	3 (17)	4 (11)	0.01* (0.92)	0.65 (0.09, 5.0)
Student	3 (17)	8 (23)	0.02* (0.87)	1.5 (0.29, 9.9)

* Kruskal–Wallis rank sum test.

† Pearson’s chi-square test.

‡ Employment status does not add up to 100% since this was a multiple-choice question and concomitant statuses could be selected.

BMI, body mass index; CI, confidence interval; IQR, interquartile range; MC, medical cannabis; n, number; OR, odds ratio.

### Medical Cannabis Cultivar and Chemovar Characteristics

**Figure 2:**
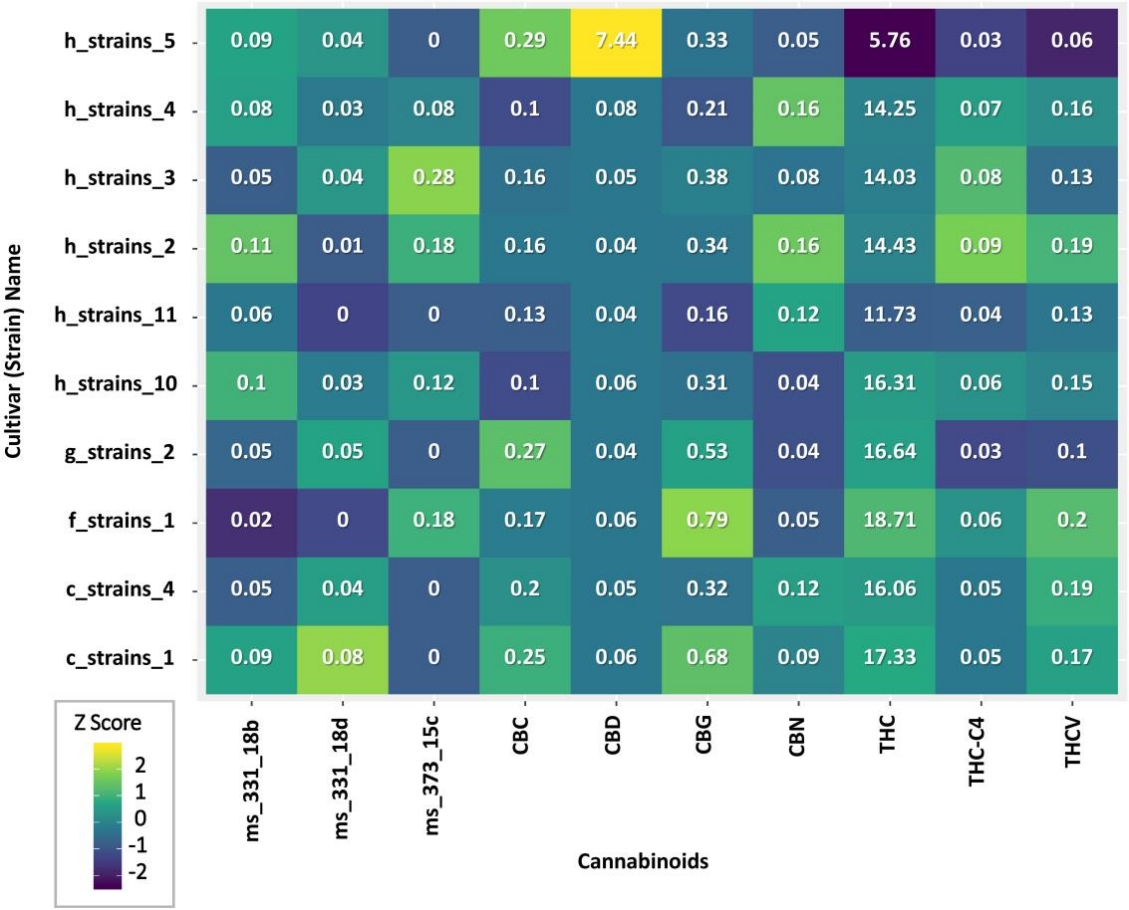
Cannabinoids Relative Dose in the Most Frequently Consumed Cultivars. Colors on the graph represent the scaled cannabinoid dose variations between cultivars; the numbers in each box represent the median concentration (%) of the specific cannabinoid within each cultivar. CBC, cannabichromene; CBD, cannabidiol; CBG, cannabigerol; CBN, cannabinol; THC, Δ-9-tetrahydrocannabinol; THC-C4, tetrahydrocannabinol-C4; THCV, tetrahydrocannabivarin.

**Figure 3:**
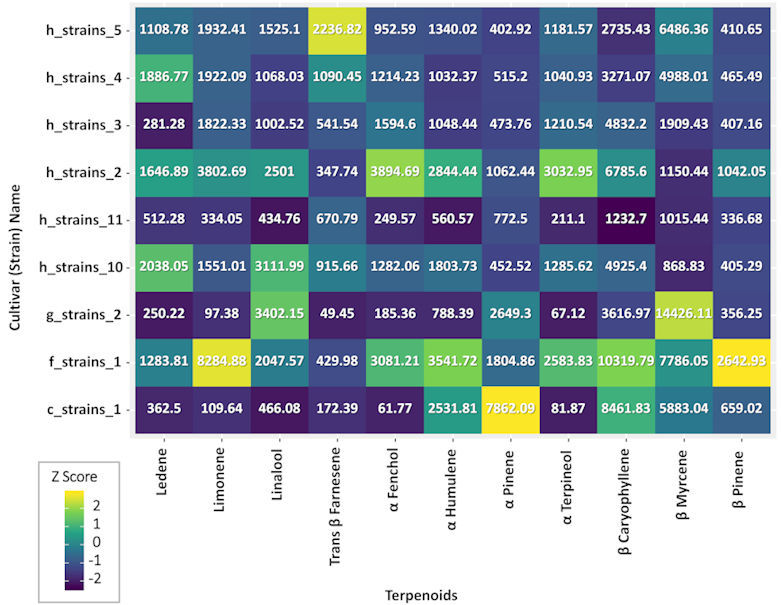
Terpenoids Relative Dose in the Most Frequently Consumed Cultivars. Colors on the graph represent the scaled terpenoid dose variations between cultivars; the numbers in each box represent the median concentration (parts per million) of the specific terpenoid within each cultivar.

Medical cannabis treatment is very complex, firstly because of the variety of cultivars in Israel (about 150 different “strain names”) and secondly because patients consume in general more than one cultivar as well as different dosages. Consequently, in the current study we have 27 unique combinations of MC cultivars. Figure 2 shows only single cultivar variability between the most prevalent phytocannabinoids in the most frequent cultivars that were consumed by the study sample, without showing the different possible combinations of chemovars used. Additionally, the most frequent cultivars and most abundant terpenoids relative content was analyzed by GC-MS/MS analysis. Figure 3 demonstrates the variability between the most prevalent terpenoids in the most frequent cultivars (without combinations) that were consumed the study sample.

**Figure 4:**
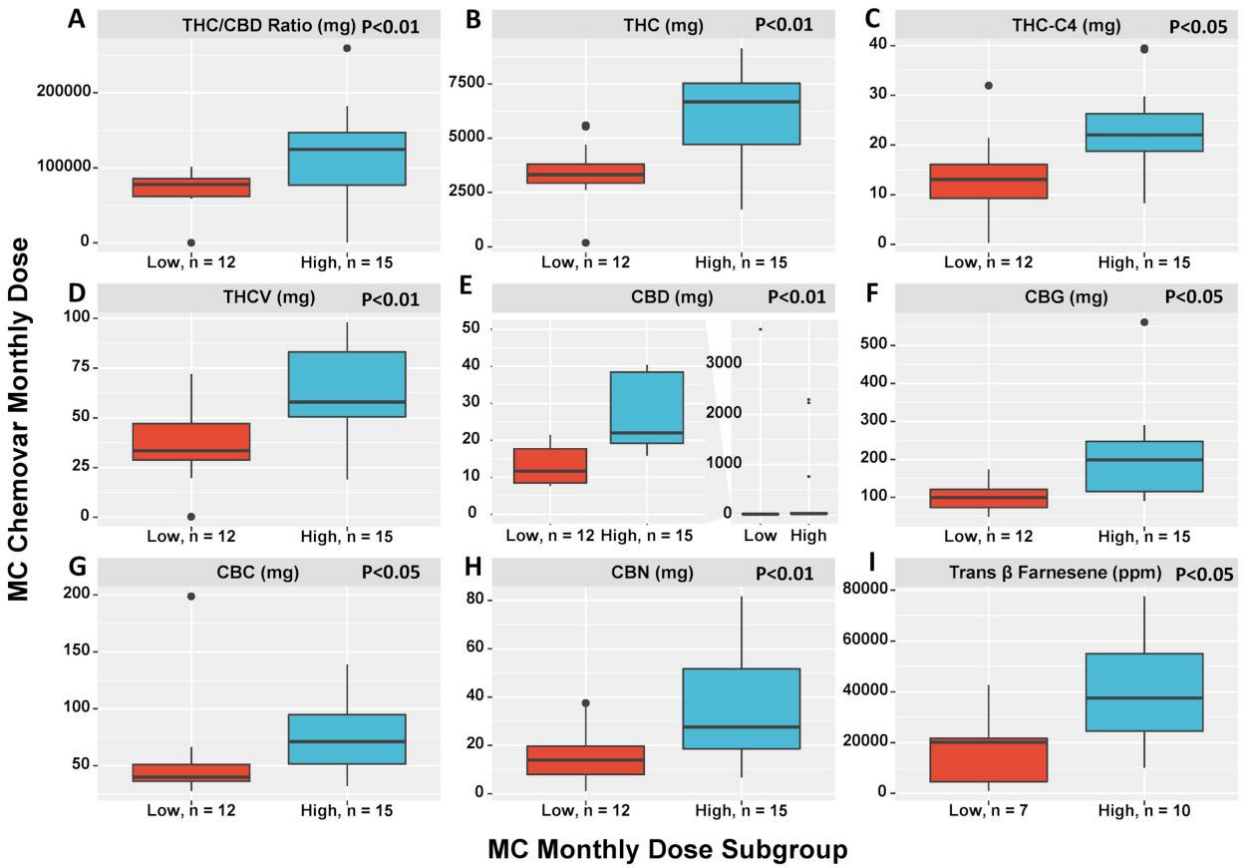
Medical Cannabis Chemovar Differences between Total MC Dose Subgroups. **A:** THC:CBD ratio dose represents the values of THC monthly dose consumption (mg) divided by the values of CBD monthly dose consumption. **B–D and F–I:** Monthly dose consumption (mg) of the mentioned MC components. **E:** Shows the difference in CBD dose consumption distribution in two ways due to extreme outliers, one without outliers (on the left of the panel) and one with all observations (on the right of the panel). CBC, cannabichromene; CBD, cannabidiol; CBG, cannabigerol; CBN, cannabinol; High, high subgroup (patients that consumed 40–70 g MC per month); Low, low subgroup (patients that consumed 20–30 g MC per month); MC, medical cannabis; mg, milligrams; ppm, parts per million; THC, Δ-9-tetrahydrocannabinol; THC-C4, tetrahydrocannabinol-C4; THCV, tetrahydrocannabivarin.

For the 27 patients that monthly MC chemovar (phyto-cannabinoids and terpenes) could be calculated for, we found that the high MC dose group consumed significantly higher amounts of the following phyto-cannabinoids compared to the low MC dose subgroup: THC, tetrahydrocannabivarin (THCV), CBD, and cannabinol (CBN) (OR −1.4, 95% CI −2.3 to −0.5; OR −1.1, 95% CI −2 to −0.25; OR −0.05, 95% CI −0.85 to 0.74; and OR −1.1, 95% CI −2 to −0.27; *P*<0.01 for all). Additionally, cannabichromene (CBC), cannabigerol (CBG), and tetrahydrocannabinol-C4 (THC-C4), and the terpene trans β farnesene (OR−0.53, 95% CI −1.3 to 0.28; OR −1.2, 95% CI −2.4 to −0.17; OR −1.2, 95% CI −2 to −0.31; and OR −1.1, 95% CI −1.9 to −0.2; *P*<0.05 for all) were also significantly higher in the high MC dose subgroup. The THC:CBD ratio dose was also higher among the high MC dose subgroup (OR −0.86, 95% CI −1.7 to −0.03; *P*<0.01) (Figure 4). Remarkably, the rest of the phyto-cannabinoids (i.e. ms_331_18b, ms_331_18d, and ms_373_15c) and terpenoids (i.e. linalool, α fenchol, ledene, limonene, sabinene, α humulene, α pinene, β caryophyllene, β myrcene, and β pinene) analyzed did not differ significantly between the MC dose subgroups. The high MC dose subgroup had a MC license for a longer duration than the low dose subgroup (OR −1.1, 95% CI −1.7 to −0.44; *P*<0.01).

### Mental Illnesses Comorbidities

Mental illnesses were reported by most (37 of 53, 70%) patients of the sample. No significant differences were found between the low and high MC dose subgroups of these comorbidities (*P*>0.05). Specifically, for the low and high MC dose subgroups, patients reported on past and current diagnosis of depression (*n*=5, 9%; and *n*=11, 21%, respectively), anxiety (*n*=7, 13%; and *n*=14, 26%, respectively), dyslexia (*n*=6, 11%; and *n*=8, 15%, respectively), as well as substance abuse (*n*=1, 2%; and *n*=6, 11%, respectively) and past alcohol abuse (*n*=1, 2% and *n*=6, 11%, respectively). A current diagnosis of bipolar disorder was reported by three patients (6%) from the high MC dose subgroup only. No antisocial disorder was reported. Current pharmaceutical treatment for the abovementioned diagnoses was reported by two (4%) patients in the low MC dose subgroup and eight (15%) in the high MC dose subgroup.

### ADHD Pharmaceutical Treatment Characteristics

**Figure 5:**
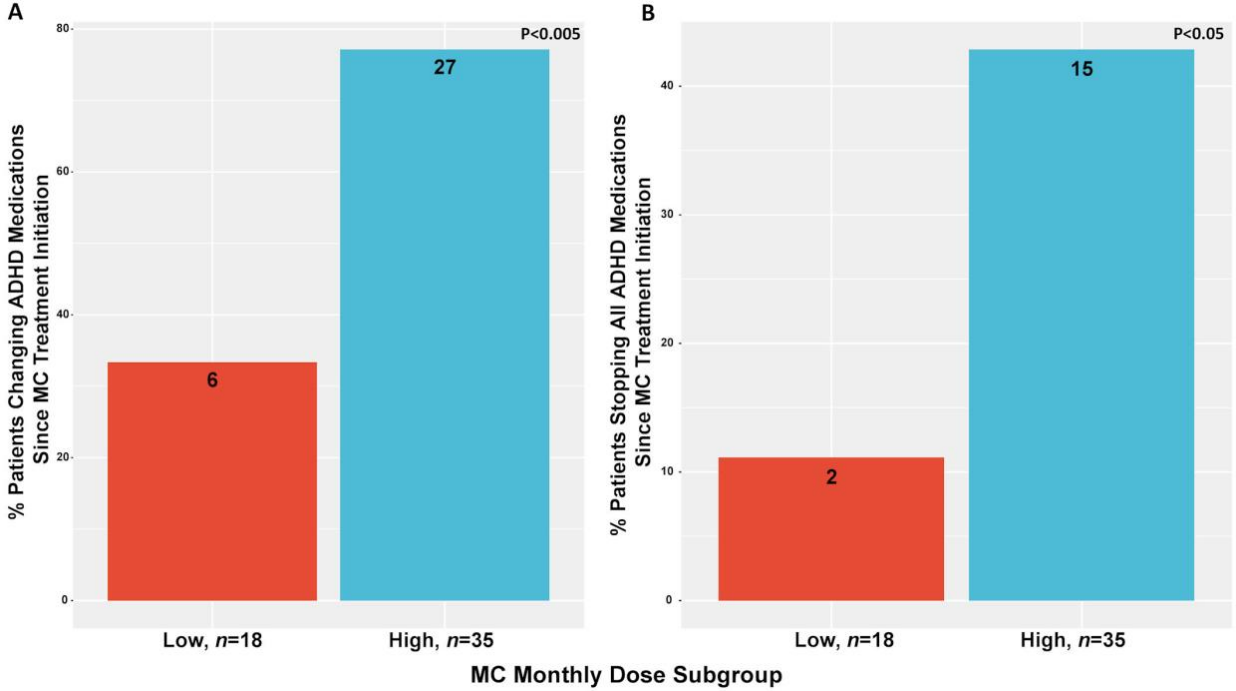
Clinical Differences between MC Dose Subgroups. **A:** MC monthly dose consumption subgroup differences in percent of change in ADHD medications since MC treatment initiation; **B:** MC monthly dose consumption subgroup differences in percent of stopping all ADHD medications since MC treatment initiation. ADHD, attention deficit hyperactivity disorder; High, high subgroup (patients that consumed 40–70 g MC per month); Low, low subgroup (patients that consumed 20–30 g MC per month); MC, medical cannabis.

A total of 16 (30%) patients reported current use of ADHD pharmaceutical medication consumption, the majority of them (*n*=10, 19%) were in the high MC dose subgroup. Specifically, the low and high MC dose subgroup reported use of methylphenidate hydrochloride (*n*=2, 4%; and *n*=1, 2%, respectively), methylphenidate hydrochloride slow release (*n*=1, 2%; and *n*=3, 6%, respectively), amphetamine and dextroamphetamine combination (*n*=1, 2%, for both), amphetamine and dextroamphetamine combination extended release (*n*=1, 2%, for both), and lisdexamfetamine dimesylate (*n*=1, 2%, for both). Two patients (4%) of the high MC dose subgroup reported use of methylphenidate hydrochloride extended release. In addition, there was an association between the high MC dose treatment and ADHD medication regimen. Specifically, the high MC dose subgroup reported significantly higher rates of changing (i.e. any type of change) their ADHD medication regimen since MC treatment initiated (OR 0.15, 95% CI 0.03 to 0.61; *P*<0.005). Furthermore, the high dose subgroup reported more on stopping all ADHD medications since MC treatment began (OR 5.8, 95% CI 1.1 to 60.0; *P*<0.05) (Figure 5).

### ADHD Symptoms Frequency

**Figure 6:**
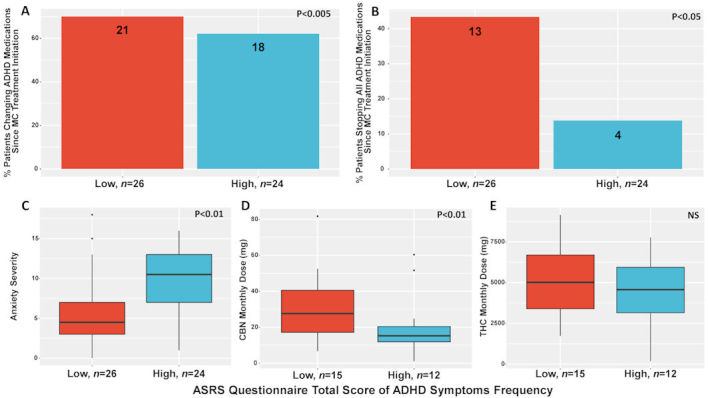
Clinical Differences between ADHD Symptoms Frequency Subgroups. **A:** ADHD symptoms severity subgroup differences in percent of change in ADHD medications since MC treatment initiation; **B:** ADHD symptoms severity subgroup differences in percent of stopping all ADHD medications since MC treatment initiation; **C:** ADHD symptoms severity subgroup differences in anxiety scores; **D:** ADHD symptoms severity subgroup differences in CBN monthly dose consumption (mg); **E:** ADHD symptoms severity subgroup differences in THC monthly dose consumption (mg). ADHD, attention deficit hyperactivity disorder; ASRS, adult ADHD self-report scale; CBN, cannabinol; High, high subgroup (patients that reported total ASRS scores ≥3.18); Low, low subgroup (patients that reported total ASRS scores ≤3.17); NS, non-significant; THC, Δ-9-tetrahydrocannabinol.

Dividing our sample by the ASRS questionnaire total score of ADHD symptoms frequency (1–5 score range, *n*=59) responses into low ASRS score (i.e. fewer ADHD symptoms, patients with ≤3.17 score, *n*=30) or high ASRS score (i.e. more ADHD symptoms, patients with ≥3.18 score, *n*=29) subgroups by our sample distribution, we found few significant differences between the subgroups (9 patients did not respond regarding analgesic medication consumption). Specifically, ADHD medications were changed more since the initiation of MC by the low ASRS score subgroup than by the high ASRS score subgroup (OR 8.6, 95% CI 1.9 to 56; *P*<0.005). The low ASRS score subgroup stopped all ADHD medications since MC treatment began more than the high ASRS score subgroup (OR 0.22, 95% CI 0.04 to 0.85; *P*<0.05). Notably, anxiety scores were higher in the high ASRS score subgroup (median 10 [IQR 7–13]) than for the low ASRS score subgroup (4.5 [3–7]) (OR −0.9, 95% CI −1.5 to −0.31; *P*<0.01). Importantly, the low ASRS score subgroup consumed higher (28 [17–41] mg) monthly CBN doses than the high (15 [12–20] mg) ASRS score subgroup (OR 0.58, 95% CI −0.24 to 1.4; *P*<0.01). However, although CBN is a metabolite of THC, we found no significant differences of monthly THC doses between the low (5000 [3400–6700] mg) and high (4600 [3200–5900] mg) ASRS score subgroups (OR 0.26, 95% CI −0.54 to 1.1; *P*=0.56) (Figure 6).

The division into low and high ASRS score subgroups was corroborated by its associations with the ADHD rating scale scores for ADHD symptom severity. Specifically, inattentiveness, compulsivity/hyperactivity, and the total questionnaire scores were higher in the high ASRS score subgroup (14 [12–20], 12 [9.2–14], and 26 [23–31], respectively) than for the low ASRS score subgroup (9 [7–11], 7 [5–9], and 16 [12–20], respectively) (OR −0.97, 95% CI −1.5 to −0.39; OR −0.7, 95% CI −1.3 to −0.14; and OR −1.1, 95% CI −1.6 to −0.49; *P*<0.001 for all). Importantly, we did not find any difference between ASRS score subgroups and ADHD rating scale regarding the monthly MC dose consumption (40 [30–50] g for both subgroups of both measures) (OR 0.05, 95% CI −0.5 to 0.61; and OR 0.11, 95% CI −0.44 to 0.66; *P*=1.0, respectively].

### MC Treatment Safety

Medical cannabis-related AEs were reported by a total of 28% (*n*=15) of the sample; AEs were not significantly different between the MC dose subgroups (*P*>0.05). Reports of AEs included central nervous system (*n*=7, 13%), gastrointestinal (*n*=7, 13%), psychological (*n*=6, 11%), cardiovascular (*n*=3, 6%), ophthalmic (*n*= 3, 6%), musculoskeletal (*n*=2, 4%), and auditory (*n*=2, 4%) AEs.

## DISCUSSION

In this study we evaluated reports of patients under MC treatment who had a comorbidity of ADHD. By calculating these patients’ monthly dose consumption of specific MC chemovar constituents, we were able to find the specific cannabinoid monthly dose in association with their ADHD symptom frequency.

Under the current regulatory framework in Israel, adjusting the dose of MC consumption is difficult. Getting approval to increase the dose may take months to years. Thus, it was not surprising to find that the higher dose subgroup had significantly longer MC license duration. In Israel, patients select cultivars that they prefer and/or that they find to be available each month, based on their approved monthly dose. Their licenses specify the condition(s) for which they are approved to take MC; however, they may self-titrate available cultivars for an effect they find therapeutic and comforting for both the conditions they are approved for and for other non-indicated comorbidities, such as ADHD. The complexity created with whole-plant products that include over 144 phyto-cannabinoids^33^ and scores of terpenoids^35^ eludes simplistic conclusions about the effect of MC on the management of ADHD symptomatology.^22^ Noteworthily, in the case report of Strohbeck-Kuehner et al.,^22^ it was suggested that cannabis treatment and synthetic THC administered to a 28-year-old man with ADHD resulted in a marked change in ADHD symptomatology without investigation of other cannabis constituents.

More recently, characterizing cultivars between THC-dominant and CBD-dominant cultivars has been used.^38^ The IMOH regulation is in the process of basing the entire Israeli MC program on this perspective, for instance.^27^ Unfortunately, this approach does not encompass the complexities of whole-plant cannabis treatment, in particular on ADHD symptom severity. Research conducted on MC treatment in ADHD is scarce and conflicting. The use of purified THC:CBD in a 1:1 ratio (nabiximols) showed no effect on ADHD symptom severity^7^; however, in a qualitative study, 25% of responses indicated that whole-plant cannabis was therapeutic for ADHD.^21^ Here, we demonstrated an association between higher CBN and lower ADHD symptoms frequency. It has been previously demonstrated that the combination of CBN and THC is associated with increased psycho-activity of THC in humans.^39^ This indicates a more complex story than simply stratifying treatment based on THC and CBD alone. Nonetheless, we did not investigate the symptom status of participants in this study prior to MC treatment initiation, so causal conclusions cannot be drawn. There is no “simplistic” method for tracking only the dominant constituents of cannabis to better understand the medical potential of a cannabis cultivar. Thus, the novel perspective of our study is extremely valuable for the MC research field.

Previous studies considered cannabis as a single product in ADHD research,^40^ disregarding its inherent complexities and variability between cultivars and combinations of cultivars, which leads to a unique amount of consumed cannabinoid and terpenoid constituents in each patient. The novelty of this study is that we did not neglect these complexities. In this study, we found that patients that consumed higher total MC monthly doses also consumed higher doses of several phyto-cannabinoids and of one terpene, but not of all of them. This finding may indicate that some constituents of the cannabis plant contribute more than others to its neurobiological effect, and may explain why some participants in our study reported substitution of conventional ADHD medications.

In this study, we demonstrated that patients treated with MC stopped their ADHD medications, especially in the high MC dose and in the low ADHD symptoms frequency subgroups. Comparably, case reports have demonstrated similar ADHD medication-sparing effects.^23^ Hence, these results might suggest that ADHD patients consume MC as a substitute treatment for their conventional ADHD treatment.

The neurobiology of ADHD is reported as being similar to other psychiatric conditions, such as bipolar disorder, which may explain the report by Katzman et al. of strong familial links between the two conditions.^41^ Similar regions and circuitry in the brain are involved in both ADHD and other psychiatric disorders, notably the limbic–cortical–striatal–pallidal–thalamic (LCSPT) circuit.^11^ Neuronal activity within the LCSPT circuits is principally glutamatergic and is modulated by the gamma-aminobutyric acid (GABA) system.^42^ This LCSPT circuitry is additionally modulated by a variety of other neuromodulators, including endocannabinoids.^43^ The majority of participants in our study reported comorbid psychiatric conditions, supporting the assertion they are linked. Anxiety was also reported here as higher by participants with high ADHD symptom frequency scores, further highlighting this link. How the endocannabinoid system may modulate the circuitry involved in both ADHD and comorbid psychiatric conditions remains to be elucidated.

Finally, the literature is rich in studies associating lower educational attainment and lower levels of employment in patients with adult ADHD.^8^ Though these topics of employment and educational achievement are outside the focus of our study, our sample of ADHD patients is unlikely to support these conclusions since our cohort falls above the reported rates by Gjervan et al.^8^ of the general population in educational achievement and education.

## LIMITATIONS

The current study has a few limitations. Firstly, the small sample size could have biased our results. Nonetheless, we used non-parametric models as is customary. Secondly, self-report bias could have occurred. However, the questionnaire was anonymous, letting patients answer with no effect on their current treatment by their physician. Thirdly, due to our study design, we did not have access to patients’ data before initiation of MC treatment, making it impossible to draw causal conclusions. Fourthly, this cohort had a diagnosis other than ADHD for which they were approved to use MC, so the data for ADHD were essentially a secondary endpoint. Nonetheless, we evaluated ADHD symptom severity by validated questionnaires.

## CONCLUSIONS

In summary, ADHD is a common psychiatric disorder in the adult population that is frequently unrecognized, under-diagnosed, and under-treated. It is often comorbid with other psychiatric disorders. Although MC is not directly indicated for ADHD, low ADHD symptom frequency and ADHD medication-sparing effects were found to be associated with MC treatment. In addition, high dosage of CBN was associated with lower ASRS, hinting at a possible combination effect in whole-plant MC treatment. Nevertheless, although we found the abovementioned association with CBN, it is minorly expressed in most MC cultivars, thus, we assume that other phyto-cannabinoids might be more essential for the effect on ADHD patients. These results, although not causal, might shed light on the potential beneficial effects of MC on ADHD symptom severity and motivate future prospective studies in order to validate our results and perhaps even consider making ADHD an approved indication for MC license in Israel in future.

## Abbreviations

ADHD: attention deficit hyperactivity disorder
AEs: adverse effects
ASRS: adult ADHD self-report scale
BMI: body mass index
CBC: cannabichromene
CBD: cannabidiol
CBG: cannabigerol
CBN: cannabinol
CI: confidence interval
GAD-7: General Anxiety Disorder-7 scale
GC-MS: gas chromatography–mass spectrometry
IMOH: Israeli Ministry of Health
IQR: interquartile range
LC-MS: liquid chromatography–mass spectrometry
LCSPT: limbic–cortical–striatal–pallidal–thalamic
MC: medical cannabis
MCU: Medical Cannabis Unit
OR: odds ratio
PSQI: Pittsburgh Sleep Quality Index
THC: Δ-9-tetrahydrocannabinol
THC-C4: tetrahydrocannabinol-C4
THCV: tetrahydrocannabivarin
UHPLC: ultra-high-performance liquid chromatography.
